# Predictors of Long-Term Outcome in Children with Hypertrophic Cardiomyopathy

**DOI:** 10.1007/s00246-015-1298-y

**Published:** 2015-11-02

**Authors:** Lidia Ziółkowska, Anna Turska-Kmieć, Joanna Petryka, Wanda Kawalec

**Affiliations:** Department of Pediatric Cardiology, The Children’s Memorial Health Institute, Al. Dzieci Polskich 20, 04-730 Warsaw, Poland; Department of Coronary Artery Disease and Structural Heart Disease, Institute of Cardiology, 04-628 Warsaw, Poland

**Keywords:** Hypertrophic cardiomyopathy, Sudden cardiac death, Cardioverter–defibrillator, Heart failure, Heart transplant, Children

## Abstract

To date limited data are available to predict the progression to end-stage heart failure (HF) with subsequent death (non-SCD), need for heart transplantation, or sudden cardiac death (SCD) in children with hypertrophic cardiomyopathy (HCM). We aimed to determine predictors of long-term outcome in children with HCM. A total of 112 children (median 14.1, IQR 7.8–16.6 years) were followed up for the median of 6.5 years for the development of morbidity and mortality, including arrhythmic and HF-related secondary end points. HF end point included HF-related death or heart transplant, and arrhythmic end point included resuscitated cardiac arrest, appropriate ICD discharge, or SCD. Overall, 23 (21 %) patients reached the pre-defined composite primary end point. At 10-year follow-up, the event-free survival rate was 76 %. Thirteen patients (12 %) reached the secondary arrhythmic end point, and 10 patients (9 %) reached the secondary HF end point. In multivariate model, prior cardiac arrest (*r* = 0.658), QTc dispersion (*r* = 0.262), and NSVT (*r* = 0.217) were independent predictors of the arrhythmic secondary end point, while HF (*r* = 0.440), LV posterior wall thickness (*r* = 0.258), LA size (*r* = 0.389), and decreased early transmitral flow velocity (*r* = 0.202) were all independent predictors of the secondary HF end point. There are differences in the risk factors for SCD and for HF-related death in childhood HCM. Only prior cardiac arrest, QTc dispersion, and NSVT predicted arrhythmic outcome in patients aged <18 years. LA size, LV posterior wall thickness, and decreased early transmitral flow velocity were strong independent predictors of HF-related events.

## Introduction

Hypertrophic cardiomyopathy (HCM) is a relatively common genetic cardiac disease, and it accounts for 42 % of childhood cardiomyopathy [14]. The severity of cardiac hypertrophy, etiology, and the clinical course of HCM in children are varied, resulting in a large spectrum of clinical and phenotypic expressions [3, 14, 16, 21, 23, 27, 35, 37]. Importantly, HCM is the most common cause of sudden cardiac death (SCD) in the young (including competitive athletes) [9, 18]. According to previous studies, the incidence of SCD in children with HCM ranges from 1 [3, 27] to 6 % [8, 32, 33]. However, limited data are available to predict which children will progress to end-stage heart failure (HF) with subsequent death or need for heart transplantation (HT) or who will die from SCD [4, 37]. Specific risk factors for SCD in adults with HCM have been proposed [6, 9, 11, 17, 38], while a few series have been focused on children [5, 15, 20, 24, 31, 33, 34, 37].

The aim of this study was to determine predictors of long-term outcome in children with HCM including known adult risk factors for SCD and previously proposed pediatric risk factors for death. Moreover, we distinguish both arrhythmic and heart failure end points to determine specific risk factors these two modes of unfavorable outcome.

## Material

### Study Patients

From May 1993 to January 2014, pediatric patients with diagnosed HCM hospitalized in the Department of Pediatric Cardiology of the Children’s Memorial Health Institute were prospectively enrolled. Criteria for inclusion in the study were age <18 years at the time of diagnosis and echocardiographic evidence of LV hypertrophy defined as a diastolic septal thickness or LV diastolic wall thickness z-score >2 (determined as more than two standard deviations from the mean value for the population corrected for body surface area (BSA) [10, 12], in the absence of hemodynamic conditions that could account for the observed hypertrophy. The study also included patients with syndrome-associated HCM. The institutional ethics committee approved this study. Informed consent was obtained from all individual participants included in the study.

### Data Collection

Patients demographics, clinical symptoms, family history of HCM and SCD, treatment strategy as well as the results of echocardiography, 12-leads ECG, 24-h Holter ECG, exercise test with assessment of blood pressure response to exercise were collected. Cardiac examination was repeatedly performed during the follow-up period. For the analysis, the most recent results or the later results before the end point were chosen. The period from the diagnosis of HCM to the most recent study ranged from 1 month to 17.3 years, median 2.7 (0.89–5.83) years. Since 2005, each patient was also screened for metabolic disorders (the metabolic tests were performed in 92 patients). Since 2010 the possibility of genetic testing occurred in individual cases. In addition, pacemaker or implantable cardioverter–defibrillator (ICD) placement, septal myectomy, and HT were recorded.

Family history of SCD (FHSCD) was defined as recommended in the literature [10]. Unexplained syncope was defined as unexplained transient loss of consciousness at or prior to first evaluation. Two-dimensional, Doppler, and M-mode echocardiography were performed at rest using standard methods. Echocardiographic measurements included septal wall thickness (SWT) and LV posterior wall thickness (LVPWT) (mm, z-score), LA size (mm, z-score), and the presence of LV outflow tract obstruction (LVOTO). LA dimension was measured at end-systole as the anteroposterior linear diameter from the parasternal long-axis view. LA enlargement was defined as z-score >2 [12]. The SWT, LWPWT in diastole, and the LA dimension were evaluated for each patient and indexed to the patient’s BSA as recommended in the literature [12]. Z-scores for the SWT, LVPWT, and the LA dimension were calculated using the formula for z-scores as reported in the literature [2] with the use of the calculator given on a website [12, parameterz.com]. Sustained monomorphic ventricular tachycardia (SVT), non-sustained ventricular tachycardia (NSVT), and an abnormal blood pressure response to exercise (ABPRE) were defined as recommended in the literature [10]. In all patients, cardiac tests results and data from the family history were collected and analyzed regarding the presence of reported adult risk factors for SCD [11, 17, 38]. In addition, in all patients previously published risk factors for death specific to the pediatric population such as SWT cutoff of >20 mm, SWT > 190 % of upper normal limit for age, QTc dispersion, LVPW-to-cavity ratio >0.30, restrictive physiology, and age at diagnosis of HCM were analyzed. Moreover, the LA size was calculated and its relevance as a risk factor for death in children with HCM was analyzed.

### Events Data

Patient events were recorded by communication with children’s parents. Medical records were reviewed after attendance in outpatient clinics or hospital stay. No patient was lost to follow-up.

The pre-specified primary end point was the composite of cardiovascular death, resuscitated cardiac arrest (CA) due to SVT or VF, appropriate ICD discharge or a HT as previously published [29]. Two separate secondary end points were predefined: A composite heart failure (HF) end point included HF-related death or HT; a composite arrhythmic end point included resuscitated CA due to SVT or VF, appropriate ICD discharge, or SCD. Patients were censored at the time of their first event or at the time of their last clinical follow-up.

### Statistics

Statistical analysis was performed using MedCalc statistical software (version 12.4, MedCalc Software, Ostend, Belgium) and SPSS software. Results are presented as median (IQR) values. Nominal variables are expressed as number of subject and the percent in the analyzed group. Comparisons of demographic and clinical characteristics between patients with and without end points were made with unpaired *t* test or Chi-square tests for continuous and categorical data, respectively.

Demographic, clinical, and scan characteristics were all first tested with univariate analysis using Cox proportional hazards model, and then all variables were taken forward to be considered for inclusion in the multivariate model. In addition, all risk factors (both proposed adult and proposed pediatric ones) were tested separately with univariate analysis for the compound arrhythmic end point and the compound heart failure end point to identify statistical significance of each variable, and then all variables were included in the multivariate model. The model was constructed with a forward selection procedure, with any variable that improved the likelihood ratio test statistic by an amount equivalent to *p* < 0.05 included. Eigenvalues and canonical correlation analysis were used to calculate the included variables participation in the variability of the response variable. The canonical variable was calculated, and the parameters the most responsible for the observed variability of the composite end point had the highest correlation value. Correlations above 0.2 indicated statistically significant parameters (correlation coefficient *r* ≥ 0.2). The statistical significance of multivariate analysis for the composite secondary arrhythmic and heart failure end point was demonstrated using an assay Pillais. Furthermore, relative risk indicators for all variables were calculated, based on the cutoff points assessed based on the comparison of frequency of distribution.

## Results

### Baseline Characteristics

A total of 112 patients with HCM were recruited between May 1, 1993 and January 31, 2014, the median age was 14.1 (7.8–16.6) years, 60 % male. Patients were followed prospectively for the median of 6.5 (2.9–9.6) years. Clinical characteristics of the patient population are presented in Table 1.

**Table 1:** Patient characteristics

Clinical parameters	Study group *n* = 112 patients
Age at clinical evaluation	
Cohort median, years (IQR)	14.1 (7.8–16.6)
Age ≤ 1	3 (2.7)
Age > 1 and ≤5	8 (7.1)
Age > 5 and ≤10	25 (22.3)
Age > 10 and <18	76 (67.9)
Age at diagnosis HCM	
Cohort median, years (IQR)	4.83 (0.54–11)
Age ≤ 1	38 (33.9)
Age > 1 and ≤5	21 (18.8)
Age > 5 and ≤10	21 (18.8)
Age > 10 and <18	32 (28.5)
Male	67 (60)
Family history	
HCM	48 (42.9)
SCD	25 (22.3)
Presenting sign/symptom	
Murmur	107 (95.5)
Chest pain	34 (30.4)
Syncope	17 (15.2)
Dyspnoea on exertion	24 (21.4)
Resuscitated sudden death	3 (2.7)
Palpitations	22 (19.6)
Heart failure (NYHA III–IV)	20 (17.9)
Follow-up, years	
Median (IQR)	6.5 (2.9–9.6)
Death	13 (11.6)
Heart failure death	7 (6.2)
SCD	6 (5.4)
ICD appropriate discharge	5 (4.5)
Resuscitated cardiac arrest	2 (1.8)
NSVT	17 (15.2)
Heart transplant	3 (2.7)
Echocardiographic data at last follow-up	
Left ventricular morphology	
Asymmetric septal hypertrophy	78 (69.6)
Concentric LV hypertrophy	31 (27.7)
Apical LV hypertrophy	3 (2.7)
Septal wall, mm	15.3 (11–21.2)
Septal wall, z-score	7.8 (4.9–15.1)
Posterior wall, mm	8.7 (7.3–11.5)
Posterior wall, z-score	1.6 (0.07–3.5)
LA size, mm	34.3 (26.4–45)
LA size, z-score	1.7 (0.6–5.1)
LVOTO ≥ 30 mmHg	28 (25)
Drug treatment	
Beta-blockers	101 (89.3)
Calcium-blockers	11 (9.8)
Antiarrhythmics (sotalol or amiodarone)	9 (8.0)
ACE inhibitors	9 (8.0)
Diuretics (furosemide or spironolactone)	34 (30.3)
Myectomy	8 (7.1)
ICD implantation	21 (18.8)
Primary prevention	15 (13.4)
Secondary prevention	6 (5.4)

Data expressed as median (IQR) or frequencies (percentages)

Genetic syndrome-associated HCM was diagnosed in eight patients (in four patients Leopard syndrome, in two patients Noonan, in one patient Costello and in one patient Kabuki syndrome). Inborn metabolic syndrome was diagnosed in four children (mitochondrial cytopatia in two patients, Pompe disease in one patient and type III glycogen storage in one patient).

### Outcomes

**Fig. 1:**
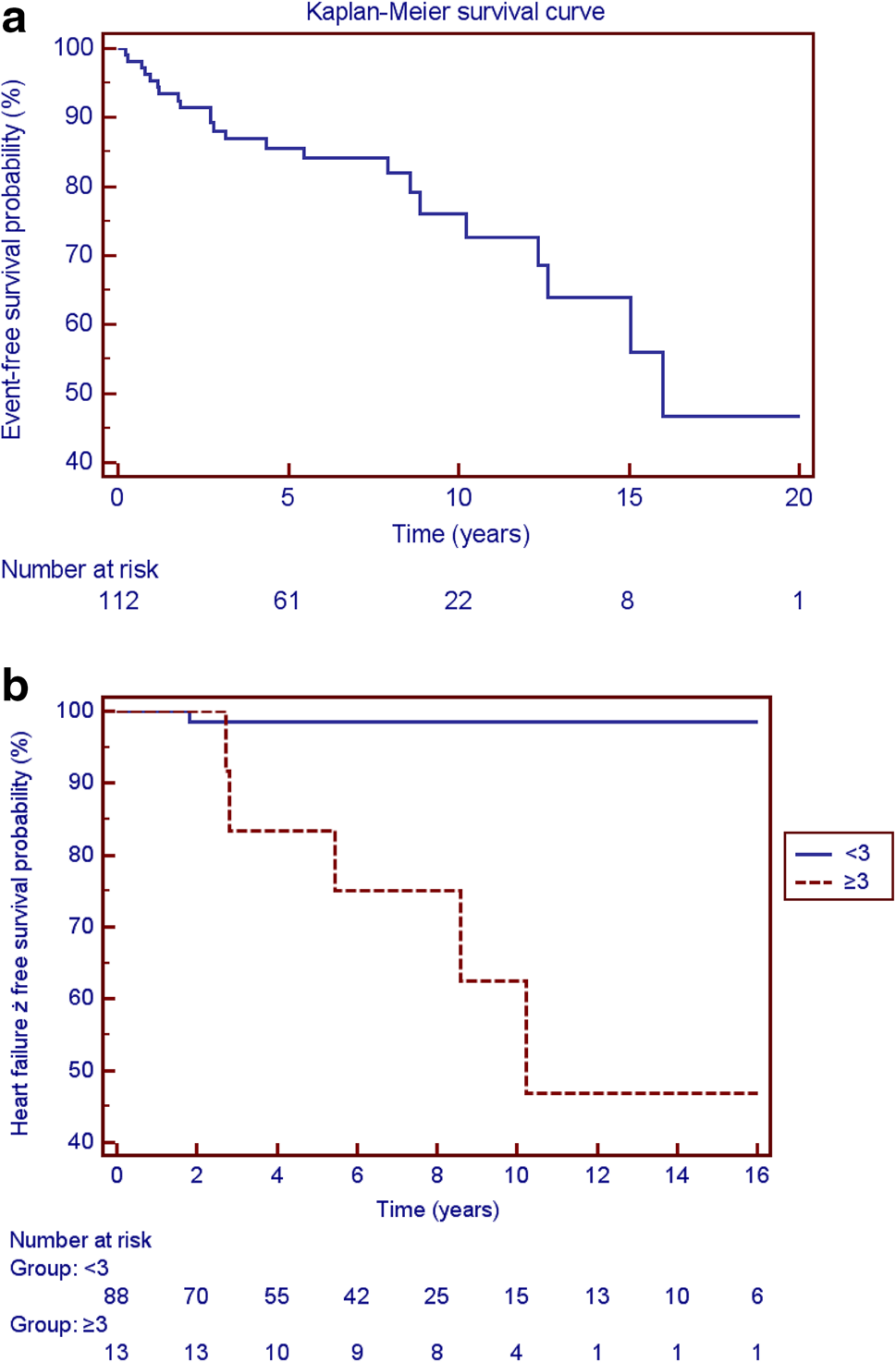
**a** Event-free survival for the study group. **b** Heart failure-free survival for patients with LA size <3 z-score and ≥3 z-score (log rank *p* < 0.0001)

During follow-up, there were 13 (11.6 %) cardiovascular deaths, six of which occurred suddenly and seven deaths were due to HF. Overall 23 (21 %) patients reached the composite primary end point (13 patients died, three children had a HT due to progressive heart failure, five children had appropriate ICD discharge and two survived resuscitated CA). Kaplan–Meier survival analysis shows 86 % event-free survival rate at 5 years and 76 % event-free survival rate at 10-year follow-up (Fig. 1a).

Thirteen patients (12 %) reached the secondary arrhythmic end point. Overall 10 of 112 patients (9 %) reached the secondary HF end point: one of 88 (1.1 %) with the LA size of <3 z-score and eight of 13 (61.5 %) with LA size of ≥3 z-score (log rank *p* < 0.0001; Fig. 1b).

**Fig. 2:**
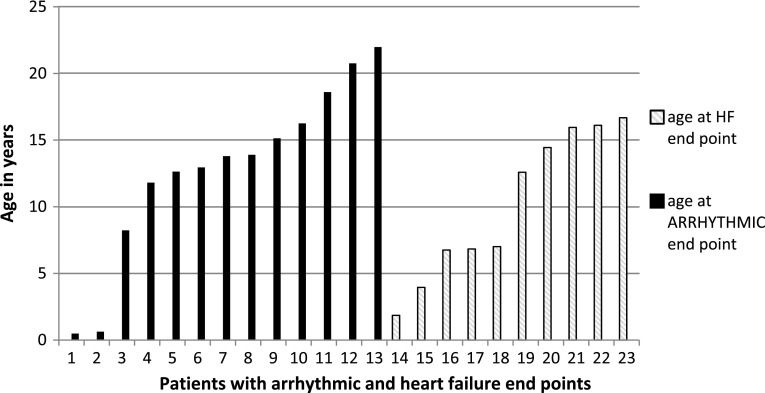
Age spectrum of arrhythmic and heart failure end points in children with hypertrophic cardiomyopathy

In the study group, no patient had both an arrhythmic and a HF end point. The median age of children at the time of arrhythmic end points was 13.8 (11.8–16.3) years, ranged from 0.5 to 22 years. The median age of patients at the time of the HF end points was 9.8 (6.8–16) years, ranged from 1.9 to 16.7 years. The age profile of patients that reached arrhythmic end point and HF end point was illustrated in a Fig. 2.

### Risk Factors for Primary and Secondary Outcomes

The frequency of reported adult risk factors for SCD and risk factors for death in children with HCM presented by other authors is summarized in Table 2. In our group of patients, no sustained ventricular tachycardia (SVT) or ventricular tachycardia (VT) not requiring resuscitation occur. In our patients, episodes of non-sustained ventricular tachycardia (NSVT) occured, which was regarded as a risk factor for sudden cardiac death, but it was not considered as a primary end point or as an arrhythmogenic secondary end point.

**Table 2:** Prevalence of adult risk factors for sudden death and previously reported pediatric risk factors for death in the study group

Risk factor (overall % of patients)	Number of observations	Patients without primary end point (*n* = 89)	Patients with primary end point (*n* = 23)	*p* value	Cutoff point value	RR (95 % CI)
Family history of SCD (22 %)	25/112	20	5	0.84	–	0.967 (0.399–2.343)
Prior cardiac arrest (3 %)	3/112	0	3	**0.004**	–	**5.450** (**3.668**–**8.098**)
Syncope (15 %)	17/112	14	3	0.99	–	0.838 (0.280–2.514)
NSVT (15 %)	17/112	11	6	0.19	–	1.972 (0.909–4.279)
Extreme LVH ≥ 30 mm (8.9 %)	10/112	8	2	0.71	–	0.971 (0.266–3.553)
ABPR to exercise (31 %)	22/70	17/62	5/8	0.11	No cutoff	0.439 (0.224–0.859)
LVOTO ≥ 30 mmHg (25 %)	28/112	20	8	0.34	No cutoff	1.600 (0.760–3.367)
LVOT gradient, mmHg	112	6.8 (5–19)	10.9 (7–31.4)	**0.04**	No cutoff	–
Age at diagnosis, years	112	5 (0.6–12)	4 (0.2–10)	0.48	No cutoff	–
SWT, mm	112	14.9 (11.1–21.2)	16.5 (10.3–20)	0.94	30	1.922 (0.292–12.657)
SWT, z-score	112	7.6 (4.7–14.9)	11.3 (5.6–16.8)	0.37	7	0.856 (0.410–1.788)
LVPWT, mm	112	8.6 (7.2–11.3)	9.6 (7.4–11.8)	0.39	10	1.315 (0.636–2.719
LVPWT, z-score		1.4 (0.1–3.2)	3 (0.1–6.3)	0.068	**4**	2.488 (1.234–5.013)
SWT of >20, mm (26.8 %)	30/112	25	5	0.54	–	0.759 (0.309–1.864)
SWT expressed as percent of 95th centile value for age	112	146.9 (116.7–219.1)	181.6 (125.8–222.2)	0.72	195	0.680 (0.330–1.402)
SWT > 190 % of upper normal limit for age (38.4 %)	43/112	32	11	0.3	–	1.471 (0.713–3.034)
QTc dispersion	112	0.04 (0.04–0.06)	0.06 (0.04–0.06)	**0.0042**	**0.055**	**3.153** (**1.506**–**6.603**)
QTc	112	0.41 (0.38–0.43)	0.42 (0.39–0.45)	0.27	0.415	1.214 (0.586–2.518)
Heart failure at presentation (17.9 %)	20/112	10	10	**0.0003**	–	**3.538** (**1.815**–**6.899**)
LA size, mm	112	34 (25.7–44.4)	39 (33–47)	0.1	38	2.496 (1.184–5.262)
LA size, z-score		1.57 (0.34–3.82)	4.8 (2.2–8.2)	**0.001**	**3**	**3.125** (**1.450**–**6.736**)
LVPWT-to-cavity ratio	112	0.22 (0.18–0.29)	0.26 (0.18–0.37)	0.15	0.6	2.650 (1.086–6.468)
LVPWT-to-cavity ratio >0.3 (26 %)	29/112	19	10	**0.03**	–	**2.202** (**1.085**–**4.468**)
E/A, cm/s	101/112	1.56 (1.25–1.9)	1.39 (1.03–1.55)	0.24	3	1.516 (0.262–8.770)
E/A, z-score		−0.72 (−1.19) to (−0.27)	−1 (−1.57) to (−0.78)	0.16	1	1.753 (0.596–5.155)
E vel, cm/s	101/112	85.2 (73.8–97)	63 (46–91)	**0.04**	70	**0.220** (**0.090**–**0.538**)
E vel, z-score		−0.5 (−1) to (0.31)	−1.94 (−2.4) to (−0.25)	**0.027**	−**1**	**0.265** (**0.107**–**0.653**)

Bold values indicate statistically significant data

Data expressed as median (IQR) or frequencies (percentages). No cutoff = frequency distribution similar, no cutoff point can be assessed

*LV* Left ventricular, *LA* left atrial, *E vel* early mitral inflow E velocity, *E/A* early mitral inflow E to late mitral inflow A ratio, *SCD* sudden cardiac death, *NSVT* non-sustained ventricular tachycardia, *LVH* left ventricular hypertrophy, *SWT* septal wall thickness, *LVPWT* left ventricular posterior wall thickness, *ABPR* abnormal blood pressure response, *LVOTO* left ventricular outflow tract obstruction

In univariate analysis previous CA (*p* = 0.004; sensitivity 13.04; 95 % CI 2.8–33.6; specificity 100; 95 % CI 95.9–100; PPV 100; 95 % CI 29.2–100; NPV 81.7; 95 % CI 73.0–88.4), the gradient value in the LVOT (*p* = 0.04; sensitivity 91.3; 95 % CI 72–98.9; specificity 43.82; 95 % CI 33.3–54.7; PPV 29.6; 95 % CI 19.3–41.7; NPV 95.1; 95 % CI 83.5–99.4), QTc dispersion (*p* = 0.0042; sensitivity 56.52; 95 % CI 34.5–76.8; specificity 77.53; 95 % CI 67.4–85.7; PPV 39.4; 95 % CI 22.7–58.2; NPV 87.3; 95 % CI 78.0–93.8), HF at presentation (*p* = 0.0003; sensitivity 43.48; 95 % CI 23.2–65.5; specificity 94.38; 95 % CI 87.4–98.2; PPV 66.7; 95 % CI 38.4–88.2; NPV 86.6; 95 % CI 78.2–92.7), LA size z-score (*p* = 0.001; sensitivity 77.78; 95 % CI 52.4–93.6; specificity 59.04; 95 % CI 47.7–69.7; PPV 29.2; 95 % CI 16.8–44.2; NPV 92.5; 95 % CI 81.8–97.9), LVPW-to-cavity ratio >0.3 (*p* = 0.03; sensitivity 43.5; 95 % CI 23.2–65.5; specificity 78.65; 95 % CI 68.7–86.6; PPV 34.5; 95 % CI 17.7–54.7; NPV 84.3; 95 % CI 74.7–91.4) and early mitral flow velocity (mitral E vel.) median value (*p* = 0.04; sensitivity 64.7; 95 % CI 38–85; specificity 84.5; 95 % CI 75–91.5; PPV 45.8; 95 % CI 25.6–67.2; NPV 92.2; 95 % CI 83.8–97.1) and z-score (*p* = 0.027; sensitivity 64.71; 95 % CI 38.3–85.8; specificity 83.33; 95 % CI 73.6–90.6; PPV 44; 95 % CI 24.0–65.5; NPV 92.1; 95 % CI 83.6–97.0) predicted primary outcome.

**Fig. 3:**
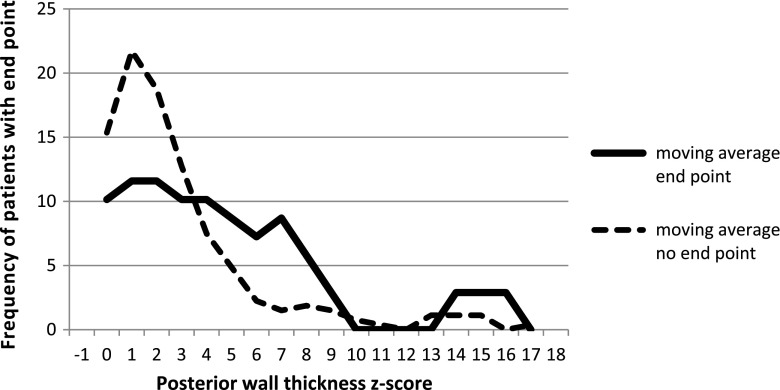
Left ventricular posterior wall thickness z-score for patients with and without primary end point, cutoff point value ≥4

**Fig. 4:**
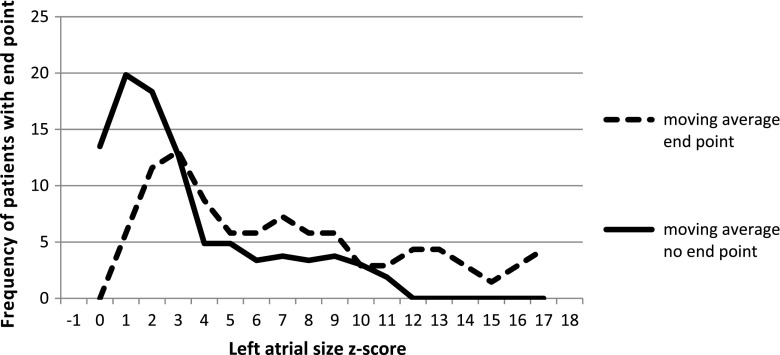
Left atrial size z-score for patients with and without primary end point, cutoff point value ≥3

**Fig. 5:**
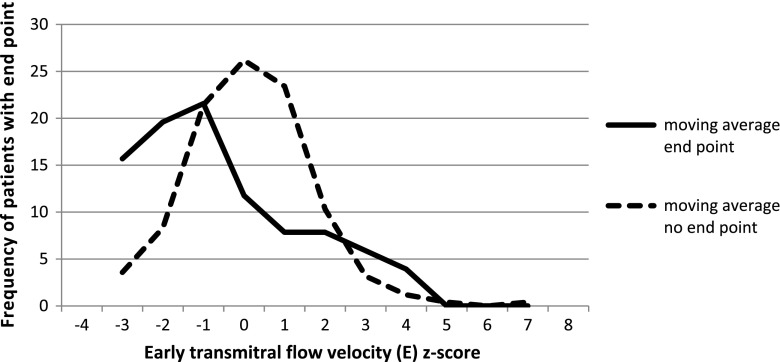
Early transmitral flow velocity (*E*) z-score for patients with and without primary end point, cutoff point value ≤−1

**Fig. 6:**
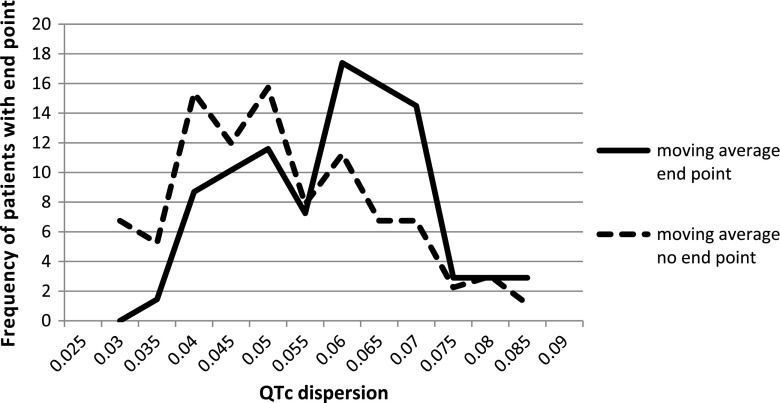
QTc dispersion for patients with and without primary end point, cutoff point value ≥0.055

We also looked for the optimal cutoff point for the risk of primary end point in our study group. The cutoff points for each risk factor for the primary end point and the value of relative risk are summarized in Table 2 and in selected Figs. 3, 4, 5 and 6.

The univariate analysis was separately performed for the compound arrhythmic end point and the compound HF end point. Only prior CA (*p* < 0.001; sensitivity 23.08; 95 % CI 5–53.8; specificity 100; 95 % CI 96.3–100; PPV 100; 95 % CI 29.2–100; NPV 90.8; 95 % CI 83.8–95.5) and QTc dispersion (*p* = 0.034; sensitivity 46.15; 95 % CI 19.2–74.9; specificity 79.8; 95 % CI 70.5–87.2; PPV 23.1; 95 % CI 8.8–44.1; NPV 91.9; 95 % CI 83.9–96.7) predicted arrhythmic secondary end point. NSVT showed a trend toward statistical significance (*p* = 0.078; sensitivity 30.77; 95 % CI 9.1–61.4; specificity 86.87; 95 % CI 78.6–92.8; PPV 23.5; 95 % CI 6.5–50.8; NPV 90.5; 95 % CI 82.8–95.6) for predicting arrhythmic secondary end point. Heart failure (*p* < 0.001; sensitivity 80; 95 % CI 44.4–97.5; specificity 93.14; 95 % CI 86.4–97.2; PPV 53.3; 95 % CI 25.7–79.5; NPV 97.9; 95 % CI 92.9–99.8), LVPWT z-score (*p* = 0.001; sensitivity 70; 95 % CI 34.8–93.3; specificity 57.84; 95 % CI 47.7–67.6; PPV 14; 95 % CI 5.8–26.7; NPV 95.2; 95 % CI 86.5–99.0), LVPW-to-cavity ratio >0.3 (*p* = 0.052; sensitivity 60; 95 % CI 26.2–87.8; specificity 77.45; 95 % CI 68.1–85.1; PPV 20.7; 95 % CI 7.8–40.1; NPV 95.2; 95 % CI 88.1–98.7), LA size (*p* = 0.041) and LA size z-score (*p* < 0.001; sensitivity 83.33; 95 % CI 35.9–99.6; specificity 93.68; 95 % CI 86.8–97.6; PPV 45.5; 95 % CI 15.6–78; NPV 98.9; 95 % CI 93.9–100.0), mitral E vel. (*p* = 0.027) and mitral E vel. z-score (*p* = 0.008; sensitivity 80; 95 % CI 28.4–99.5; specificity 86.46; 95 % CI 78–92.6; PPV 23.5; 95 % CI 6.5–50.8; NPV 98.8; 95 % CI 93.5–100.0) predicted secondary HF end point in univariate model.

In the multivariate canonical analysis, only prior CA (*r* = 0.658), OTc dispersion (*r* = 0.262), and NSVT (*r* = 0.217) were explaining the variance of the arrhythmic secondary outcome. For the secondary HF outcome heart failure (*r* = 0.440), LA size z-score (*r* = 0.389), LVPWT z-score (*r* = 0.258) and mitral E vel. z-score (*r* = 0.202) explained the variance of the end point in multivariate canonical analysis.

## Discussion

### Population Characteristics

The clinical characteristic of our study group remains comparable to the previously described cohorts [1, 3, 5, 24, 27]. In the study group 21 % of patients reported fatigue or dyspnea on exertion, which is in concordance with previously reported rates (14–24 %) [5, 27]. Similar to other studies 30 % of children complained of chest pain and 20 % complained of palpitations [3]. Should be noted that as many as 18 % of children presented with symptomatic HF (NYHA class III and IV) which exceeds the frequency presented by other authors [15, 31]. It should be stressed that children under 1 year of age at diagnosis of HCM constituted 34 % of our group. Furthermore eight (7 %) patients had coexisting genetic syndrome (Noonan, Leopard, Costello and Kabuki), and four patients (3.5 %) had inborn metabolic disorders (mitochondrial cytopathy, Pompe and type III glycogen storage disease). Among eight patients with an underlying genetic syndrome, two (25 %) children reached the composite heart failure end point (HF-related death in one patient with Noonan and HT in one patient with Leopard syndrome). Out of four children with inborn metabolic disorders, in one (25 %) patient a composite arrhythmic end point occurred (resuscitated CA in child with mitochondrial cytopathy).

Alike in previous studies [5, 7] in 3 % of patients, the prior resuscitated sudden cardiac death was their presenting symptom. It should be stressed that almost half of the children (43 %) had a family history of HCM in at least one family member, while in other studies familial incidence of HCM ranged from 17 to 43 % [3, 5, 11]. Also, there was a higher incidence of FHSCD due to HCM (22 % in the analyzed group of children vs 17 % in other study) [5].

### Left Atrial Size

This is the first prospective study to demonstrate the value of LA size on echocardiography to independently predict outcome in children with HCM. Previously, marked LA enlargement has been reported to predict outcome in adult patients with HCM [36] but not in children. In the study by Nistri et al. LA size of more than 48 mm (the 75th percentile) increased the risk of HF-related death by 3.1 but was unassociated with SCD [26]. Other study demonstrated the potential clinical importance of LA volume index in pediatric HCM as a marker of the severity of underlying diastolic dysfunction, symptom score and decreased exercise capacity [22]. In the study by Maskatia et al. [20] restrictive physiology, defined as LA enlargement, E/E′ ratio at least 10 or E/A ratio >3 correlated with all-cause cardiac death or aborted SCD in childhood HCM. The results of our study demonstrated that LV diastolic dysfunction expressed by decreased mitral E vel. was associated with HF-related unfavorable prognosis. In our study LA size was an independent predictor of both the primary end point and secondary HF-related outcome. Just like in the study by Nistri et al. [25], the results of our research have shown that LA enlargement predicted HF-related unfavorably outcome and was not associated with SCD. Patients with LA size of <3 z-score were much less likely to suffer from HF-related end points than patient with LA enlarged above 3 z-score.

### Adult Risk Factors

We found that among known adult risk factors previous CA was an independent predictor of both primary end point and secondary arrhythmic outcome in children with HCM. It is worth emphasizing the fact that NSVT was a statistically significant predictor of the arrhythmic secondary outcome in multivariate analysis in the studied group. The results of study by Monserrat et al. are similar, showing that the risk of SCD is significantly higher in younger (< 30 years of age) patients with NSVT on Holter ECG [22]. It should be emphasized that the current European Society of Cardiology (ESC) guidelines include NSVT into a major risk factors for SCD in children with HCM [10]. The fact that FHSCD was not a risk factor for SCD in childhood HCM in the studied group deserves a special attention. The later seems to be very important because, in the opinion of some authors, it would be reasonable to implant an ICD with only one risk factor, for example FHSCD [11]. Although risk factors in adult have been well described [9, 11, 17, 38], it seems that they suboptimally differentiate patients at high and low risk of SCD [28, 30]. Therefore, the ICD implantation should be planned with great caution, especially in pediatric population [13, 19]. The current ESC guidelines [10], in addition to recommended so far major risk factors for SCD, take into account additional risk factors for SCD in adults such as LA size, patient age, and LVOTO. These parameters allow more individualized risk prediction on which clinical decisions can be made.

### Pediatric Risk Factors

Very few series focused on the evaluation of risk factors for death in children with HCM. Predictors such as hypertrophy (wall thickness z-score >6) and ABPR to exercise [5] or a SWT more than 190 % of upper limit for age, and/or QRS amplitude sum of more than 10 mV on limb lead ECG [31, 33], SWT cutoff of >20 mm [31], QTc dispersion [37] have been previously reported as independent risk factors for HF-related death or SCD in small populations. The study by Ostman-Smith, which showed that risk factors for SCD and for HF-related death are not the same in childhood HCM, demonstrated that Noonan syndrome and generalized cardiac hypertrophy with a LVPW-to-cavity ratio >0.30 were risk factors for HF-related death [31]. The results of our study are consistent with the above cited publication [31]. Risk factors for SCD and HF-related death differ in our pediatric population with HCM. Heart failure, LVPWT, LA size, and decreased mitral E vel. were independent predictors of the secondary HF outcome in multivariate analysis but were unassociated with SCD. Interestingly, in our pediatric cohort LVPW-to-cavity ratio >0.3 demonstrated to predict secondary HF outcome in univariate analysis but not in the multivariate model.

In the study by Moak et al. [24] traditional adult risk factors such as FHSCD, VT on Holter and exercise-induced hypotension did not predict events, whereas SWT at least 20 mm and inducible VT on electrophysiological study did. In another study by Yetman et al. [37] increased QTc dispersion on baseline ECG was significantly associated with an increased risk of SCD what was also demonstrated in our pediatric cohort. QTc dispersion predicted both primary end point and secondary arrhythmic outcome both in univariate and multivariate model. In our studied group risk factors for SCD and secondary arrhythmic outcome such as prior CA, QTc dispersion, and NSVT were not associated with HF-related end point.

### Mortality and Mode of Death

We found an 86 % event-free survival rate at 5 years and 76 % event-free survival rate at 10-year follow-up. The overall mortality in our cohort was 11.6 %, which is similar to that of 11.4 % (11 deaths in 96 patients) reported by Decker et al. [5]. In our study HF-related deaths occurred almost as frequent as SCD (6.2 vs 5.4 %), what remains in keeping with previous reports [5, 31, 37]. Interestingly, the HF—free survival rate was 92 % at 5-year follow-up and 85 % at 10 years, which is in keeping with previous data [33]. The results of our study demonstrated that patients with arrhythmic end point were older than children with heart failure end point.

## Conclusions

The risk factors for sudden death and for heart failure-related death are different in childhood HCM. Only prior CA, QTc dispersion and NSVT predicted arrhythmic outcome in patients of <18 years of age. LA size, LVPWT, and decreased mitral E velocity were strong independent predictors of heart-failure-related events. The prediction of clinical outcome in children with HCM still remains a challenge and should be the subject of large-scale multicenter trials.

No association between the medications and end points has been found in our study. It seems reasonable to maintain the current clinical practice with special caution in patients with risk factors for both heart failure and arrhythmic end points.

### Limitations

The main limitation of our study is a lack of genetic testing, which has become more widely available just recently. The risk factors for both primary and secondary end points can possibly vary in different genetic conditions. Further studies with genetics provided are required.

Secondly, we included patients with an underlying syndrome what made our study group less homogenous.
